# The role of innovation and entrepreneurship in promoting common prosperity in China: Empirical evidence from a two-way fixed effects model

**DOI:** 10.1371/journal.pone.0295752

**Published:** 2023-12-13

**Authors:** Tingting Zhang, Wenxuan Wang, Chunyu Li, Xiaofan Wang

**Affiliations:** 1 School of Economics, Capital University of Economics and Business, Huaxiang, Fengtai District, Beijing, 100070, China; 2 School of Economics and Finance, Huaqiao University, Fengze District, Quanzhou, Fujian, 362021, China; 3 College of Mathematics and Statistics, Hebei University of Economics and Business, Xinhua District, Shijiazhuang, Hebei, 050061, China; 4 Department of Finance, Shandong Technology and Business University, Laishan District, Yantai, Shandong, 264005, China; Amirkabir University of Technology (Tehran Polytechnic), ISLAMIC REPUBLIC OF IRAN

## Abstract

Common prosperity stands as a pivotal concept and objective within China’s socialism with distinctive characteristics, serving as a fundamental assurance and basis for ensuring its people’s happiness and comprehensive development. This research employs a Coupled Coordination Degree Model to construct a common prosperity Index using data from China between 2010 and 2020. The study investigates the influence of innovation and entrepreneurship on common prosperity while examining the regulating roles played by the government and market during this process. The outcomes demonstrate that innovation substantially facilitates the realization of common prosperity. The relationship between entrepreneurship and common prosperity follows a positive "U"-shaped curve, where entrepreneurship significantly contributes to common prosperity upon reaching a particular scale. Further investigations reveal heterogeneity in the impact of innovation and entrepreneurship on common prosperity. Specifically, innovation significantly contributes to common prosperity in the northern regions, whereas entrepreneurship has a noteworthy impact on common prosperity in the southern regions. Moreover, it is worth noting that both innovation and entrepreneurship have a significant influence on common prosperity in areas characterized by low economic development levels and a scarcity of fixed capital. The fiscal effects of the government attenuate the promoting effect of innovation on common prosperity but enhance the adverse influence of entrepreneurship. On the contrary, market mechanisms mitigate the negative impact of entrepreneurship on common prosperity. Consequently, achieving common prosperity requires strengthened regional innovation cooperation, encouraging advanced regions to lead underdeveloped regions, and leveraging the regulatory roles of both the government and the market, thus progressing gradually towards common prosperity.

## 1. Introduction

Common prosperity is a universal objective economies pursue, making it a global issue. China has been committed to achieving common prosperity for all its citizens: common prosperity is essential to socialism. China’s modernization adheres to the development ideology centered on the people. The nation conscientiously addresses regional disparities, urban-rural gaps, and income distribution imbalances, promoting social fairness and justice. The gradual realization of common prosperity for the entire population is a crucial goal while resolutely preventing the occurrence of extreme polarization. The 19th National Congress report underscored the ambitious goal of "common prosperity for all." The sixth plenary session of the 19th CPC Central Committee further emphasized implementing the new development concept, fostering high-quality development, and advancing common prosperity. The 20th CPC National Congress in 2022 reiterated the importance of achieving common prosperity. It recognized "enriching the people’s spiritual world and achieving common prosperity for all" as a fundamental requirement for socialist modernization. Common prosperity, as an essential requirement and aspiration of socialism, reflects the fundamental principles of China’s socialist development [1]. However, achieving the goal of common prosperity may be a long and formidable undertaking [2]. Hence, addressing the pressing and significant question of how to achieve common prosperity in the new era and fully establish a socialist, modernized, and prosperous nation is essential.

As China’s economic development enters a new phase, it faces both the need to enhance economic quality and efficiency and the challenges of downward pressure. The country advocates for innovation and entrepreneurship to invigorate economic development [3]. Innovation, the core engine driving high-quality economic development under the new development paradigm, represents the primary driving force for progress. Emphasizing innovation-driven development is crucial for China to overcome the middle-income trap [4,5]. It can foster increased urban and rural incomes by raising total factor productivity and optimizing industrial structure [6]. Moreover, innovation in areas such as education, healthcare, and transportation can also elevate the well-being of diverse societal groups, promoting shared prosperity and advancing social equity [7]. However, some scholars posit that technological advancements brought about by innovation constitute a significant driver of structural unemployment. Suppose the quality of the labor force within society fails to align with the production demands. In that case, innovative enterprises may reduce their labor requirements [8], posing a challenge to achieving common prosperity.

The vitality of entrepreneurship can ignite the dynamism of new industries and business models, thereby providing fresh impetus for achieving common prosperity [9]. Stimulating the entrepreneurial vigor of the entire population is an effective means to address social conflicts, improve people’s livelihoods, and achieve social equity [10]. Achieving this goal can be accomplished through various pathways, such as the entrepreneurship-driven poverty reduction effect [11,12], job creation, wealth accumulation leading to further prosperity, and the enhancement of the quality and development opportunities of the impoverished [13], all of which contribute to narrowing income disparities [14] and, thereby, facilitate the realization of common prosperity.

However, due to regional disparities in economic development levels and industrial structures, the impact of entrepreneurship varies. Research suggests central and eastern China’s entrepreneurial activities can significantly boost income levels and promote common prosperity [15]. However, in Western regions, entrepreneurship can have a reverse impact on income, hindering the achievement of common prosperity.

Based on the above analysis, innovation and entrepreneurship are crucial in achieving common prosperity. Therefore, what impacts do innovation and entrepreneurship have on common prosperity? Will these impacts vary due to regional disparities, differences in economic development levels, and variations in fixed capital stock? Can the government and market play a regulatory role in these impacts? This paper aims to investigate these questions.

## 2. Literature review and research hypotheses

### The essence of common prosperity

The theoretical connotations of common prosperity have been continuously refined and possess distinct characteristics of the contemporary era. While numerous scholars have extensively examined the topic of common prosperity, the academic community has yet to establish a universally acknowledged theoretical framework [16].

Some scholars define common prosperity as "achieving equality by compensating and rectifying institutional factors that lead to inequality, allowing all citizens to have equal opportunities and capabilities to participate in high-quality economic and social development, and sharing the benefits of economic and social progress" [17]. Other scholars argue that common prosperity is "a dynamic optimization and realization of distribution goals between extreme states of the income distribution (i.e., egalitarianism and polarization)." Its achievement relies on the development of productive forces and the support of socialist public ownership [18]. Some scholars propose a "three-step" approach to achieving common prosperity: the first step involves lifting a certain segment of the population out of poverty, the second step entails the prosperous leading the less fortunate, and the third step focuses on achieving common prosperity for all citizens [19]. Based on this, three fundamental implications of common prosperity are derived: firstly, ensuring the safety net of common prosperity, providing appropriate relief to the impoverished, and allowing them to be in a position to pursue prosperity and not remain in absolute poverty, secondly, employing the strategy of "prosperity leading to prosperity," where the prosperous individuals extend a helping hand to the less fortunate, giving them hope of attaining prosperity, making common prosperity more readily acceptable, thirdly, striving for common prosperity that satisfies all members of society, allowing every citizen to reach a state of relative affluence, breaking free from poverty, and maintaining a level of relative abundance at or above the moderate level. Some scholars also interpret the terms "common" and "prosperity" separately, suggesting that prosperity reflects the high level of development in productive forces, while shared defines the socialist nature of prosperity. It reflects how members of society share in the outcomes of development, embodying the nature of socialist production relations. People consider poverty and dual stratification incompatible with socialism because achieving "common" prosperity is the only way to attain true socialism [20]. In this sense, prosperity includes material abundance and spiritual richness [21].

### Innovation and common prosperity

The achievement of common prosperity is inseparable from innovation, and technological innovation is a crucial cornerstone for enhancing comprehensive national strength and international competitiveness. It also serves as an essential force in providing a solid material foundation for realizing common prosperity. Firstly, technological innovation is beneficial for enhancing total factor productivity [22], promoting industrial structure upgrading [23], and driving high-quality economic development [24], thereby enlarging the "economic pie" and increasing the national income level. Secondly, technological innovation exhibits diminishing marginal returns, and its impact may vary between economically developed and underdeveloped regions, which contributes to narrowing the wealth gap between regions. Technological innovation generates employment opportunities through various channels, leading to employment compensation effects [25,26] reducing income disparity among different population groups. Thirdly, technological innovation can effectively lower the cost of social infrastructure construction, improve the working environment for laborers, reduce harm to their health, and decrease medical expenses, which contributes to promoting harmonious development and facilitates the equitable sharing of benefits in areas such as education, healthcare, and environmental protection that result from the development [27].

However, due to the significant heterogeneity of innovation [28], it only sometimes positively impacts achieving common prosperity. For instance, technological innovation can accelerate industrial agglomeration. However, excessively high industrial concentration may contradict the principles of resource endowment and comparative advantage, resulting in a loss of production efficiency [29] and detrimental to national income. Furthermore, technological innovation may exhibit substitution effects concerning employment [8,30,31], hindering efforts to narrow the wealth gap. Additionally, due to differences in developmental stages and policies, the changes in industrial structure induced by innovation may not necessarily lead to environmentally friendly transformations, potentially causing environmental pollution [28]. Consequently, this could adversely affect public health and hinder the equitable sharing of development outcomes among all citizens.

Based on the analysis above, we propose hypothesis 1.

**Fig 1:**
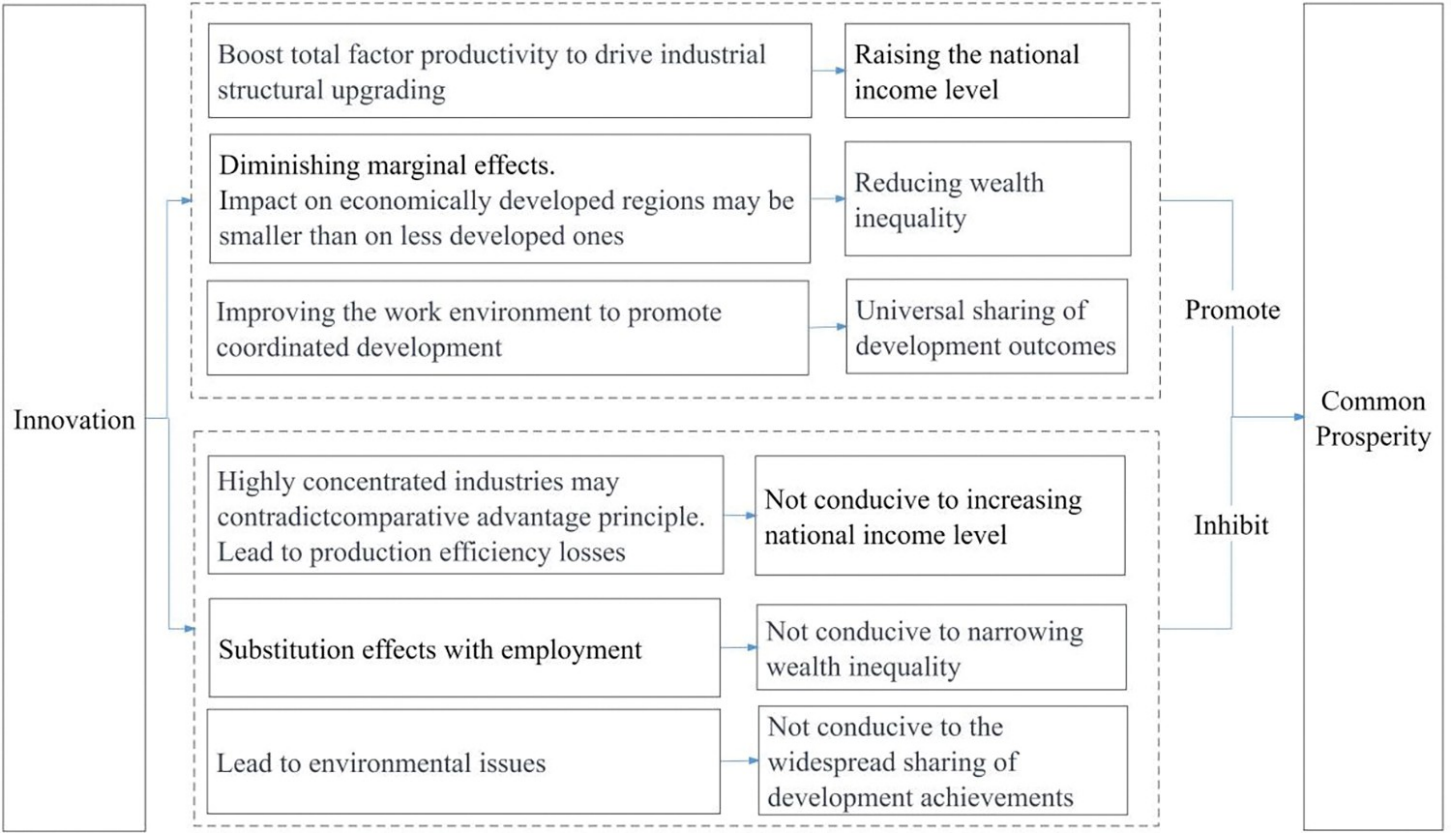


**Fig 1** illustrates the impact of innovation on common prosperity.

Hypothesis 1: Innovation significantly influences common prosperity. This influence may manifest in positive and negative aspects, with the net effect depending on the strength of these opposing influences.

### Entrepreneurship and common prosperity

Entrepreneurial activities are the intrinsic driving force behind economic growth [10] and significantly influence the realization of common prosperity. Firstly, entrepreneurial activities can promote industrial upgrading [32], providing impetus for economic growth and raising overall income levels. The return of migrant workers to their hometowns for entrepreneurship is also a key driver for increasing rural income [33]. Secondly, entrepreneurial activities have a broad impact [34], creating more employment opportunities [35] and contributing to poverty alleviation efforts [36–39], thus reducing income disparities among different population groups. Thirdly, entrepreneurship can extend social security systems, such as healthcare and education, to agricultural laborers [3], bringing previously jobless laborers into new startup enterprises and enabling them to enjoy social security benefits.

However, not all entrepreneurial activities can effectively contribute to common prosperity, and entrepreneurship may not necessarily reduce poverty levels significantly [40]. Regional disparities in economic development limit entrepreneurial resources in China’s central and western regions. As a result, the impact of entrepreneurial activities on income improvement in these regions is far less significant compared to the eastern regions. In some cases, entrepreneurship has been found to reduce income significantly [15]. Whether entrepreneurship can promote common prosperity also depends on the type of entrepreneurship. Survival-oriented entrepreneurship, instead of opportunity-oriented entrepreneurship, may only sustain rural households’ basic livelihoods without further narrowing the urban-rural wealth gap [41].

Additionally, entrepreneurship, which brings higher income to entrepreneurs, may lead to widening income disparities among residents [42]. Moreover, individuals with different initial wealth endowments may choose different occupations. Those with higher initial wealth endowments are likelier to opt for entrepreneurship to achieve higher income, while people experiencing poverty are more inclined to choose wage work. An oversupply of labor in the labor market can result in lower incomes for people experiencing poverty who choose wage work, leading to an expansion of income disparities [43].

Based on the analysis above, we propose hypothesis 2.

**Fig 2:**
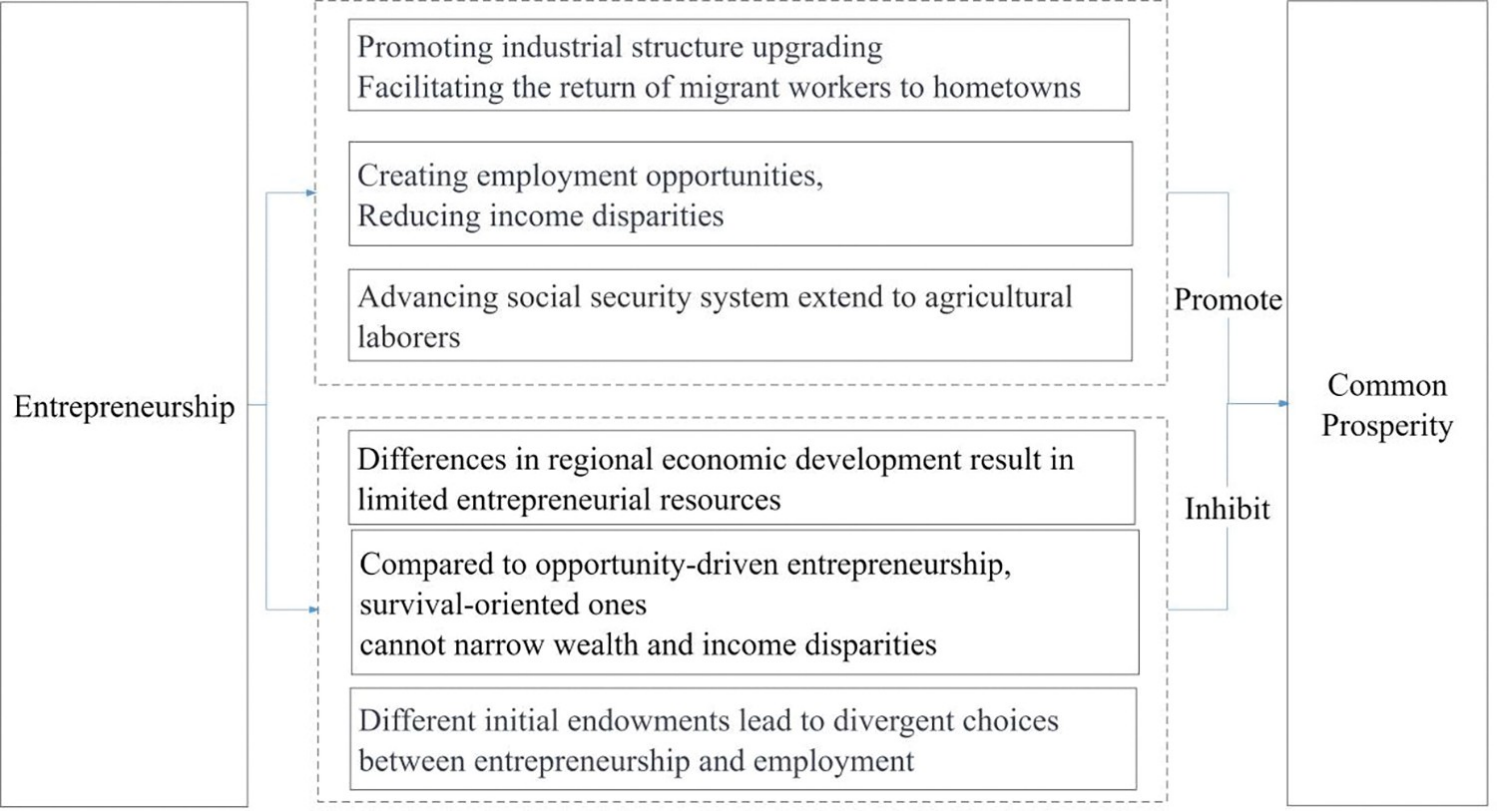


**Fig 2** illustrates the impact of entrepreneurship on common prosperity.

Hypothesis 2: Entrepreneurship will significantly impact common prosperity, which may manifest positively and negatively. The net effect depends on the strength of the positive and negative influences.

### Literature review

A review of existing literature reveals that scholars have long been engaged in research on common prosperity, with a relatively larger body of theoretical studies focusing on the meaning and mechanisms of common prosperity. In recent years, many researchers have shown interest in constructing common prosperity indices to quantify this concept and explore its realization pathways using econometric models. To achieve common prosperity, innovation and entrepreneurship play significant roles.

Based on the existing literature, innovation, for instance, can foster common prosperity by enhancing total factor productivity, optimizing industrial structures, and improving the job environment. However, it may also adversely affect common prosperity, such as reduced production efficiency, environmental pollution, and job displacement due to high industrial concentration. Similarly, entrepreneurship can contribute to achieving common prosperity by reducing poverty and extending social security systems to impoverished groups. However, it is also possible that income inequality may widen due to differences in the types of entrepreneurship and resource endowments, which could be detrimental to common prosperity. Hence, the academic community has differing views on whether innovation and entrepreneurship can promote common prosperity. This paper aims to analyze this issue and enrich existing research findings.

Based on this, the present study constructs a common prosperity index using a coupling coordination model. It employs a fixed effects model to investigate the impacts of innovation and entrepreneurship on common prosperity. The potential marginal contributions are as follows:

First, the common prosperity index is developed based on three dimensions, wealth, equality, and sharing, to study the effects of innovation and entrepreneurship on common prosperity. The findings reveal that innovation can promote common prosperity, while entrepreneurship contributes to common prosperity only after reaching a particular scale, which differs from previous research conclusions. Second, the study analyzes the effects of innovation and entrepreneurship on common prosperity from the perspectives of regional disparities, economic development levels, and heterogeneity in fixed capital stock. Third, the research examines the moderating effects of government and the market on the impact of innovation and entrepreneurship on common prosperity, thus enriching the existing body of research on this topic.

## 3. Model and variables

### Model building

Firstly, to analyze the impact of innovation and entrepreneurship on common prosperity, refer to existing literature, we construct the econometric model in Eq (1) for empirical analysis [9].


$${Cpi}_{it}=\alpha_{0}+\alpha_{1}{Ino}_{it}/{Entre}_{it}+\alpha_{k}{Controls}_{it}+\mu_{i}+\delta_{t}+\varepsilon_{it}$$ (1)

The subscripts *i* and *t* represent provinces (or municipalities) and years, respectively. The dependent variable is the Common Prosperity Index (*Cpi*), and the key explanatory variables are the levels of innovation (*Ino*) and entrepreneurship (*Entre*). The control variables (*Controls*) include the advancement of industrial structure (*TS*), financial development (*Fin*), foreign direct investment utilization (*FDI*), population density (*Rmd*), urbanization rate (*Ur*), and human capital (*Hz*). The variable *α*_0_ represents the constant term, *μ*_*i*_ denotes individual fixed effects, *δ*_*t*_ denotes time-fixed effects, and *ε*_*it*_ represents the random disturbance term.

Secondly, to examine the moderating effects of local government fiscal and marketization levels, interaction terms between innovation, entrepreneurship, and variables representing the extent of local government fiscal regulation and marketization index are added to Eq (1). Subsequently, the interaction terms’ constituent variables are centered. The econometric model is presented as Eqs (2) to (5).

Subsequently, we refer to existing literature to examine the moderating effects of local government fiscal policies and the level of marketization [44]. In Eq (1), we introduce interaction terms between innovation, entrepreneurship, the degree of local government fiscal regulation, and the marketization index. We also apply centering to the sub-variables of the interaction terms. The econometric model is presented as Eqs (2) through (5) in the following manner:

$$Cpi_{it}=\beta_{0}+\beta_{1}Ino_{it}+\beta_{2}Gov_{it}+\beta_{3}Ino_{it}\times Gov_{it}+\beta_{k}Controls_{it}+\mu_{i}+\delta_{t}+\varepsilon_{it}$$ (2)

$$Cpi_{it}=\beta_{0}+\beta_{1}Ino_{it}+\beta_{2}Mar_{it}+\beta_{3}Ino_{it}\times Mar_{it}+\beta_{k}Controls_{it}+\mu_{i}+\delta_{t}+\varepsilon_{it}$$ (3)

$$Cpi_{it}=\beta_{0}+\beta_{1}Entre_{it}+\beta_{2}Gov_{it}+\beta_{3}Entre_{it}\times Gov_{it}+\beta_{k}Controls_{it}+\mu_{i}+\delta_{t}+\varepsilon_{it}$$ (4)

$$Cpi_{it}=\beta_{0}+\beta_{1}Entre_{it}+\beta_{2}Mar_{it}+\beta_{3}Entre_{it}\times Mar_{it}+\beta_{k}Controls_{it}+\mu_{i}+\delta_{t}+\varepsilon_{it}$$ (5)

Where *Gov*_*it*_ represents the variable of the extent of local government fiscal regulation, and *Mar*_*it*_ denotes the degree of marketization, the meanings of other variables are the same as in Eq (1).

### Variables

#### The dependent variable

Common Prosperity Index (*Cpi*). We can measure common prosperity using the overall common prosperity index and the degree of sharing [45]. The overall index measures the gap between China and developed countries regarding per capita national income, per capita wealth holdings, per capita material wealth holdings, and overall labor productivity levels. We assess the degree of sharing from three aspects: regional, urban-rural, and group disparities. Some scholars evaluate common prosperity from three dimensions [16], development, sharing, and sustainability, by constructing three primary indicators, 14 secondary indicators, and 81 tertiary indicators. Other scholars emphasize that common prosperity should achieve wealth and equality, with both factors being equally important and requiring dynamic coordination and organic integration [46]. They propose constructing a common prosperity index system based on the perspectives of income and equality.

This study considers common prosperity a complex system composed of three subsystems: wealth, equality, and sharing [47]. These three subsystems are interrelated and engage in a dynamic developmental process. Because the forces acting upon the wealth, equality, and sharing subsystems are not necessarily uniform and may even be contradictory, the evolution of these subsystems does not inherently progress from disorder to order. The orderly development of the wealth subsystem does not inevitably lead to the orderly development of the equality and sharing subsystems and vice versa. Only through the organic unity and coordinated development of these three subsystems can the goal of achieving common prosperity be realized. From the perspective of coupled coordination, we can understand common prosperity as achieving high coordination between the wealth, equality, and sharing subsystems when they are highly coupled. In summary, this paper assesses common prosperity by constructing a coupled coordination model with specific indicators outlined in Table 1.

**Table 1:** Evaluation system for the common prosperity index.

Subsystem	Primary index	Attribute
**The wealth subsystem (U1)**	Per Capita Gross Domestic Product (GDP)	+
	Per Capita Disposable Income of Urban Residents	+
	Per Capita Disposable Income of Rural Residents	+
**The equality subsystem (U2)**	Urban-Rural Income Ratio	-
	Gini Coefficient of Income Disparity by Industry	-
	Gini Coefficient of Income Disparity	-
	Unemployment Rate	-
**The sharing subsystem (U3)**	The Proportion of Livelihood-Oriented Fiscal Expenditure	+
	Average Enrollment in Higher Education Institutions per 100,000 People	+
	Number of Health Technical Personnel per 10,000 People	+

The wealth subsystem (*U1*) includes economic development and income level indicators. The equality subsystem (*U2*) includes indicators such as the urban-rural income gap, industry income gap, and income gap among different groups. The sharing subsystem (*U3*) includes public financial expenditure, education, and medical resources indicators.

Before calculating the index of common prosperity, performing a power transformation on the original indicators is necessary. Let *x*_*ij*_ be the original indicator, and Eq (6) gives its power transformation:

$${x^{\prime }}_{ij}=\{\begin{array}{c} (x_{ij}-M_{ij})/(N_{ij}-M_{ij}),x_{ij}\;exhibits\;positive\;power\;effect\\ (N_{ij}-x_{ij})/(N_{ij}-M_{ij}),x_{ij}\;exhibits\;negative\;power\;effect \end{array}$$ (6)

*N*_*ij*_ represents the maximum value of *x*_*ij*_, and *M*_*ij*_ represents the minimum value of *x*_*ij*_.

After performing the power transformation on the original indicators, the comprehensive ranking of each subsystem can be obtained through weighting (with weights determined by the entropy method), which allows for the construction of the coupling degree models for the three subsystems, as shown in Eq (7):

$$C=\sqrt[{3}]{(U1*U2*U3)/{\left(\frac{U1+U2+U3}{3}\right)}^{3}}$$ (7)

*C* represents the coupling degree among the three subsystems (reflecting the degree of interdependence and mutual constraint between the systems).

Next, we construct the coordination degree *T* among the three subsystems based on the coupling degree, which reflects the degree of coordination between the systems, as shown in Eq (8):

$$\{\begin{array}{c} D=C*T\\ T=\alpha_{1}U1+\alpha_{2}U2+\alpha_{3}U3 \end{array}$$ (8)

Where *α*_1_+*α*_2_+*α*_3_ = 1, based on existing literature, equal weights are assigned to *U1*, *U2*, and *U3*. *D* represents the common prosperity index, reflecting the degree of mutual coordination and development among the three subsystems.

#### The core explanatory variable

Innovation (*Ino*) and Entrepreneurship (*Entre*). Existing literature mainly measures innovation through the levels of innovation input and output. Considering that invention patents better reflect the quality of innovation, the number of applied and authorized invention patents is used as a measure [48]. As for entrepreneurship, it is represented by private enterprises and self-employed individuals [49].

#### The moderator variable

Local government fiscal regulation (*Gov*). The ratio of the sum of personal income tax and corporate income tax to GDP represents the local government fiscal regulation level.

Degree of marketization (*Mar*). The Comprehensive Marketization Index, published by the National Economic Research Institute, measures the degree of marketization [50].

#### The control variable

Other factors also influence common prosperity. In order to better analyze the impact of innovation and entrepreneurship on common prosperity and reduce estimation bias caused by omitted variables, this study introduces a series of control variables, such as industrial structure [51].

(1) Industrial Structure (*TS*): The optimization and upgrading of industrial structure can provide sustained momentum for common prosperity, facilitating the expansion of the "economic pie" and affecting wealth and income distribution, as well as promoting coordinated development between urban and rural areas, thereby contributing to the equitable distribution of the "economic pie." We represent the level of industrial structure upgrading as 1 × the proportion of GDP contributed by the primary industry + 2 × the proportion of GDP contributed by the secondary industry + 3 × the proportion of GDP contributed by the tertiary industry [52,53].

(2) Financial Development (*Fin*): The development of the financial industry is conducive to better serving the real economy, promoting high-quality economic development, and achieving common prosperity. The level of financial development is represented as the ratio of total loans and deposits to GDP [54].

(3) Foreign Direct Investment (*FDI*): Based on the successful experience of reform and opening-up, promoting economic development and achieving common prosperity requires adherence to openness to the outside world. Foreign direct investment has been crucial in driving wealth accumulation and technological progress in China. The level of foreign direct investment is represented as the logarithm of actual foreign direct investment [55].

(4) Population Density (*Rmd*): Population growth inherently signifies an expansion of the labor force—a factor that, as posited by the production function, fosters economic growth and correlates intimately with broadening the economic landscape. However, from another perspective, population growth also affects factor distribution related to the equitable distribution of the "economic pie." Therefore, population density has a significant impact on achieving common prosperity. We represent population density as the population per square kilometer [56].

(5) Urbanization Rate (*Ur*): Urbanization involves the cross-regional movement of factors of production, which affects various aspects of economic activities and plays an essential role in achieving common prosperity. We represent the level of urbanization as the ratio of the urban population to the total population [57].

(6) Human Capital (*Hz*): According to economic growth models, human capital can effectively avoid the problem of diminishing marginal returns of physical capital and significantly impact achieving common prosperity. We represent the level of human capital as the logarithm of the number of students enrolled in regular higher education per ten thousand people [58,59].

Table 2 shows the definitions, symbols, and indicators for each variable.

**Table 2:** Variable definitions.

Variables		Symbols	Indicator explanations
**Dependent Variable**	Common Prosperity Index	Cpi	Constructed from the three sub-dimensions of wealth, equality, and sharing using the coupling coordination model
**Core Independent Variable**	Innovation Level	Ino	In logarithmic form, the number of domestic invention patent applications granted
	Entrepreneurship Level	Entre	In logarithmic form, the number of employees in private enterprises and self-employed individuals
**Moderating Variable**	Local government fiscal and taxation regulation level	Gov	The ratio of personal income tax and corporate income tax to GDP
	Marketization level	Mar	Measured from five aspects: the relationship between government and market, the development of the non-state-owned economy, the development of product markets, the development of factor markets, and the development of market intermediary organizations and legal institutions
**Control Variable**	Industrial Structure	TS	1× the proportion of GDP from the primary industry + 2× the proportion of GDP from the secondary industry + 3× the proportion of GDP from the tertiary industry
	Financial Development	Fin	The ratio of total loans and deposits to GDP
	Foreign Direct Investment	FDI	Actual foreign direct investment is converted into Chinese Yuan based on the average USD exchange rate to CNY each year, in logarithmic form.
	Population Density	Rmd	The number of people per square kilometer
	Urbanization Rate	Ur	The ratio of urban population to total population
	Human Capital	Hz	Represented by the number of students enrolled in regular higher education institutions per ten thousand people, in logarithmic form, included in the model

**Data source and descriptive statistics of variables.** Due to data availability, this study is based on the annual balanced panel data of 30 provinces, autonomous regions, and municipalities in China (excluding Hong Kong, Macao, Taiwan, and Tibet) from 2010 to 2020. We construct a two-way fixed effects model to investigate how innovation and entrepreneurship impact common prosperity. The original data of income Gini coefficient are sourced from the statistical yearbooks of various provinces and municipalities. Other relevant data are obtained from the National Bureau of Statistics, "The China Statistical Yearbook," the China Economic Information Network (CEInet) database, and the EPS database. The data are deflated to 2010 prices to remove the influence of price level changes. Table 3 presents descriptive statistics for the relevant variables.

**Table 3:** Descriptive statistics of main variables.

Variables		Sample size	Mean	Standard deviation	Minimum value	Maximum value
**Dependent Variable**	Cpi	330	0.5594	0.1300	0.08	0.93
**Core Independent Variable**	Ino	330	8.0720	1.4560	3.71	11.17
	Entre	330	6.4955	0.9071	4.10	8.62
**Moderating Variables**	Gov	330	0.0175	0.0115	0.01	0.06
	Mar	330	6.7146	2.0284	2.33	12.00
**Control Variables**	TS	330	2.4375	0.3979	1.53	4.59
	Fin	330	3.3285	1.0903	1.68	7.58
	FDI	330	5.4383	1.6992	-1.22	7.72
	Rmd	330	0.2876	0.1167	0.08	0.58
	Ur	330	0.5836	0.1253	0.34	0.90
	Hz	330	7.8243	0.2965	6.99	8.73

Table 3 shows that the average level of common prosperity is 0.5594, with a minimum value of 0.08 and a maximum value of 0.93. A significant disparity between the minimum and maximum values indicates a substantial imbalance in common prosperity among different provinces, autonomous regions, and municipalities (excluding Hong Kong, Macau, Taiwan, and Tibet). The process of achieving common prosperity still has a long way to go.

The mean value of the innovation variable is 8.0720, with a minimum of 11.17 and a maximum of 3.71. The standard deviation is 1.4560, suggesting a substantial variation in innovation levels among different provinces, autonomous regions, and municipalities (excluding Hong Kong, Macau, Taiwan, and Tibet). As for the entrepreneurial variable, the mean value is 6.4955, with a minimum of 4.1 and a maximum of 8.62. The standard deviation is 0.9071, indicating a certain level of variation in entrepreneurial levels among different provinces, autonomous regions, and municipalities (excluding Hong Kong, Macau, Taiwan, and Tibet). However, in comparison to innovation, the entrepreneurial volatility is relatively lower.

## Empirical results and the analysis

### Basic results analysis

Table 4 presents the baseline model regression results on the impact of innovation and entrepreneurship on common prosperity. Models (1) and (2) represent the effect of innovation on common prosperity, validating hypothesis 1. Meanwhile, models (3) and (4) represent the effect of entrepreneurship on common prosperity, aimed at validating hypothesis 2.

**Table 4:** The baseline regression results.

Variables	Cpi (Robust Standard Errors)	Cpi (Ordinary Standard Errors)	Cpi (Robust Standard Errors)	Cpi (Ordinary Standard Errors)
	(1)	(2)	(3)	(4)
**Ino**	0.0750**	0.0750**		
	(2.0018)	(2.2234)		
**Entre**			-0.1053*	-0.1053***
			(-1.7941)	(-2.6768)
**TS**	0.1772*	0.1772**	0.1494*	0.1494*
	(1.9622)	(1.9886)	(1.6962)	(1.7360)
**Fin**	0.0396	0.0396	0.0272	0.0272
	(1.4264)	(1.4387)	(0.9986)	(0.9850)
**FDI**	-0.0230**	-0.0230*	-0.0110	-0.0110
	(-2.4345)	(-1.8284)	(-1.0884)	(-0.8885)
**Rmd**	0.0264	0.0264	0.0280	0.0280
	(0.1511)	(0.1774)	(0.1635)	(0.1889)
**Ur**	1.4483***	1.4483***	1.5717***	1.5717***
	(3.3926)	(3.1329)	(3.4495)	(3.4639)
**Hz**	-0.4177*	-0.4177***	-0.3455	-0.3455***
	(-1.7133)	(-4.2343)	(-1.6403)	(-3.4647)
**Province**	Yes	Yes	Yes	Yes
**Year**	Yes	Yes	Yes	Yes
**_Cons**	5.4981***	5.4981***	6.2207***	6.2207***
	(3.6704)	(6.8589)	(3.5596)	(8.0581)
**N**	330	330	330	330
**R** ^**2**^	0.9327	0.9327	0.9332	0.9332

Note: t-values in parentheses

* p < 0.1

** p < 0.05

*** p < 0.01.

Regarding the core explanatory variables, the estimated coefficient of the innovation variable is statistically significant at the 5% level, with a value of 0.0750, indicating that innovation significantly promotes the achievement of common prosperity. On the one hand, innovation activities can improve productivity, enhance total factor productivity, and stimulate rapid and sustainable economic development, leading to an improved standard of living and enlarging the "economic pie," thereby raising the level of prosperity for the entire population. On the other hand, innovation provides effective means for resource allocation, fostering fairness and equity in income distribution, ensuring that the benefits of development are shared by all, significantly benefiting underdeveloped regions and low to middle-income groups. Innovation is inclusive in improving the living standards of these regions and groups, thereby reducing regional and income inequalities. Technological innovation also leads to product and service upgrades, enhancing people’s quality of life and meeting their increasing aspirations for a better life [60].

The estimated coefficient of the entrepreneurship variable is significant at the 10% level, with a value of -0.1053, indicating that entrepreneurship activities, to some extent, hinder the achievement of common prosperity. This finding contrasts with the conclusion drawn in many studies that entrepreneurship has poverty reduction effects [2,61]. The underlying reasons can be attributed to several factors. On the one hand, resources are scarce, leading to a situation where only individuals with certain initial wealth accumulation possess the capital and ability to choose entrepreneurship. Successful entrepreneurs then accumulate wealth through entrepreneurial activities, while low-income individuals may only access lower wages through regular work, thus further widening income disparities among different groups.

On the other hand, developed regions might have more attractive entrepreneurship policies and environments, inspiring more people with entrepreneurial vision and energy, leading to a more active entrepreneurial climate and widening the wealth gap between different regions. We might observe entrepreneurship activities being unfavorable for achieving common prosperity when the scale of the entrepreneurial group is relatively small. At this stage, only a minority may experience wealth accumulation through entrepreneurship, contributing to an initial concentration of wealth. However, as the scale of entrepreneurship expands, the increase in entrepreneurial enterprises reduces labor supply and increases labor demand, leading to rising wage levels. Consequently, the income gap between entrepreneurs and wage earners gradually narrows [3], and the negative impact of entrepreneurship on common prosperity might transform into a positive one (further verification of this will be discussed in the following sections).

In the control variables, the coefficient of the industrial structure upgrading variable is significant, at least at the 10% level and positive. Industrial upgrading can further facilitate the national economic cycle, increase the potential economic growth rate, provide a more solid material foundation for common prosperity, and promote wealth and income redistribution, thus helping to narrow income disparities. The coefficient of financial development on common prosperity is positive but insignificant. This could be due to distortions in the allocation of financial resources in some regions, where an overall increase in financial development fails to promote common prosperity significantly. The coefficient of foreign direct investment (FDI) on common prosperity is negative. Foreign investment contributes to China’s wealth accumulation and technological progress, effectively addressing regional capital shortages and promoting economic development. However, different regions attract foreign investment to varying degrees, with developed areas being more attractive than less developed ones, which widens the wealth gap between regions. The coefficient of population density on common prosperity is positive but insignificant. This may be attributed to the fact that, on the one hand, higher population density indicates an abundant labor force, which can contribute to economic growth and the expansion of the "cake." On the other hand, a large population may result in a smaller "cake" per capita, ultimately not significantly affecting common prosperity. The coefficient of urbanization rate on common prosperity is significant at the 1% level.

On the one hand, urbanization involves the migration of labor and other production factors from rural areas to cities. Cities have advantages in terms of infrastructure, market environment, and institutions compared to rural areas, which can enhance productivity and promote economic growth, contributing to increased prosperity. On the other hand, the urban-rural income gap is a crucial factor in wealth disparity. An increase in the urbanization rate helps narrow the income gap between urban and rural areas, promoting common prosperity.

The coefficient of the impact of human capital levels on common prosperity is negative. This may be because while human capital can promote economic growth, it tends to concentrate more in developed regions. Moreover, regions with higher levels of human capital demand higher wage levels, leading to increased income inequality, which is detrimental to achieving common prosperity. Additionally, there are significant disparities in education and human capital levels between urban and rural areas. Thus, it is reasonable to consider agricultural laborers as low-skilled workers and non-agricultural laborers as high-skilled workers [62]. Factors such as technological advancements that promote economic development tend to be endogenously biased towards high-skilled workers, leading to a technology premium and an increase in income for high-skilled workers [63]. Consequently, this exacerbates income disparities between high-skilled and low-skilled workers and between urban and rural areas, ultimately impacting the realization of common prosperity.

### Robustness test

This paper mainly examines the impact of innovation and entrepreneurship on common prosperity. The empirical results show that innovation can significantly promote common prosperity, while entrepreneurship negatively impacts common prosperity. In order to validate the robustness of the empirical findings, this paper conducts robustness tests from three aspects: changing the sample period, changing the sample scope, and lagging the core explanatory variables by one period. The results of the robustness tests are presented in Table 5, and the regression model coefficients are estimated using robust standard errors.

**Table 5:** Robustness tests.

Variables	Cpi	Cpi	Cpi	Cpi	Cpi	Cpi	Cpi	Cpi
	(1)	(2)	(3)	(4)	(5)	(6)	(7)	(8)
**Ino**	0.0917**		0.0667*					
	(2.1242)		(1.7354)					
**Entre**		-0.1108*		-0.1164*				
		(-1.8636)		(-1.8632)				
**L.lno**					0.0466**	0.0859*		
					(2.0717)	(1.7405)		
**L.Entre**							-0.0852**	-0.0713*
							(-2.3025)	(-1.8329)
**TS**	0.2188**	0.1687*	0.1661*	0.1523*		0.1491		0.1022
	(2.2004)	(1.8024)	(1.8815)	(1.7225)		(1.4533)		(1.1774)
**Fin**	0.0585*	0.0409	0.0431	0.0319		0.0432		0.0311
	(1.9157)	(1.3818)	(1.4432)	(1.0884)		(1.4186)		(1.1042)
**FDI**	-0.0294***	-0.0187*	-0.0192*	-0.0083		-0.0209***		-0.0095
	(-2.7378)	(-1.8131)	(-1.7456)	(-0.6681)		(-2.6496)		(-0.7717)
**Rmd**	0.0292	0.0417	0.0304	0.0674		-0.0334		-0.0197
	(0.1683)	(0.2419)	(0.1654)	(0.3545)		(-0.1705)		(-0.1320)
**Ur**	1.6055***	1.7583***	0.9091	1.1214*		0.8562**		1.0895**
	(3.3095)	(3.3424)	(1.3987)	(1.7187)		(2.3992)		(2.4139)
**Hz**	-0.4494	-0.3563	-0.4677*	-0.3873*		-0.2677		-0.2132**
	(-1.6245)	(-1.4918)	(-1.8333)	(-1.7898)		(-1.0000)		(-2.0450)
**Province**	Yes	Yes	Yes	Yes	Yes	Yes	Yes	Yes
**Year**	Yes	Yes	Yes	Yes	Yes	Yes	Yes	Yes
**_Cons**	5.2863***	6.0962***	6.2275***	6.6998***	4.0094***	4.6671***	4.9717***	5.3864***
	(3.1471)	(3.1083)	(3.6082)	(3.5621)	(20.5061)	(2.9049)	(21.2828)	(6.3941)
**N**	300	300	286	286	300	300	300	300
**R** ^**2**^	0.9315	0.9317	0.9267	0.9279	0.9442	0.9477	0.9448	0.9471

Note: t-values in parentheses

* p < 0.1

** p < 0.05

*** p < 0.01.

#### 4.2.1. Changing the sample period

We excluded the data for the year 2020 due to the influence of short-term economic shocks and other factors on the economy. We adjusted the sample period to 2010–2019 and conducted a new regression. Table 5 presents the results for models (1) and (2). From the results in the table, it can be observed that the coefficient of innovation on common prosperity is significant at the 5% level, indicating that innovation can promote common prosperity. On the other hand, the coefficient of entrepreneurship on common prosperity is significant at the 10% level and negative, suggesting that entrepreneurship has a certain negative impact on common prosperity. After changing the sample period, the robustness test results are consistent with the baseline regression results.

#### 4.2.2. Changing the sample scope

Among the provincial-level administrative units, municipalities directly under the central government (Beijing, Tianjin, Chongqing, and Shanghai) hold a special status and receive policy preferences. Hence, we excluded the data for these four municipalities and conducted a new regression. Table 5 presents the results for models (3) and (4). From the results in the table, it can be observed that the coefficient of innovation on common prosperity is significant at the 10% level, indicating that innovation can significantly promote common prosperity. On the other hand, the coefficient of entrepreneurship on common prosperity is significant at the 10% level and negative, suggesting that entrepreneurship has a certain negative impact on common prosperity. After changing the sample scope, the robustness test results are consistent with the baseline regression results.

#### 4.2.3. Core explanatory variable lagged by one period

The impact of innovation and entrepreneurship on common prosperity may exhibit a time lag. Therefore, this study introduces the lagged one-period core explanatory variables into the model for robustness testing, and the results are presented in models (5) to (8) in Table 5. Model (5) represents the impact of the one-period lagged innovation variable on common prosperity without including control variables, while model (6) represents the impact after incorporating control variables. The results in columns (5) and (6) show that, with or without control variables, the coefficient of the one-period lagged innovation variable on common prosperity is significant, at least at the 10% level, indicating that innovation can promote common prosperity. Model (7) represents the impact of the one-period lagged entrepreneurship variable on common prosperity without control variables, and model (8) represents the impact after including control variables. From the results in columns (7) and (8), it can be observed that, with or without control variables, the coefficient of the one-period lagged entrepreneurship variable on common prosperity is significant, at least at the 10% level and negative, suggesting that entrepreneurship to some extent hinders the realization of common prosperity. The robustness test results for the one-period lagged core explanatory variables are consistent with the baseline regression results.

The above tests show that the estimation results of the baseline model are robust, and the research conclusions are relatively reliable.

### Nonlinear effect analysis

The baseline model regression results indicate that entrepreneurship negatively impacts common prosperity to a certain extent. On the one hand, this negative effect is attributed to entrepreneurship activities widening income disparities and hindering the achievement of common prosperity. On the other hand, the scale of the entrepreneurial population still needs to be bigger to achieve the goal of common prosperity for the entire population. As the size of the entrepreneurial group grows, it may contribute to common prosperity.

When the number of entrepreneurial enterprises is low, their ability to address employment is limited, and the labor market experiences an oversupply of labor, leading to lower wage levels for workers [3]. Although entrepreneurs create wealth through their business activities and provide employment opportunities and wages for those choosing to work, individuals seeking employment at this stage have lower bargaining power in the labor market, resulting in lower wages. During this phase, entrepreneurial income has a minimal impact on income distribution and can even widen income disparities, thereby hindering the achievement of common prosperity. On the other hand, when the number of entrepreneurial enterprises is high, the labor market faces a labor supply shortage, possibly leading to excess demand. This situation empowers workers with higher bargaining power, increasing labor income to reach an equilibrium between supply and demand. During this phase, increased entrepreneurial activity significantly reduces income disparities among different groups, thus realizing common prosperity.

Based on the above analysis, this study incorporates the quadratic term of the entrepreneurship level in the baseline model for empirical analysis. The results are presented in Table 6, where model (1) represents the impact of the entrepreneurship level and its quadratic term on common prosperity without controlling for other variables; model (2) includes control variables but does not control for province fixed effects and time fixed effects; model (3) controls for province fixed effects but not time fixed effects; and model (4) controls for both province fixed effects and time fixed effects.

**Table 6:** Nonlinear effect of entrepreneurship level on common prosperity.

Variables	Cpi	Cpi	Cpi	Cpi
	(1)	(2)	(3)	(4)
**Entre**	-0.3372**	-0.4459***	-0.4522***	-0.3665**
	(-2.3776)	(-2.7363)	(-2.7729)	(-2.4074)
**Entre_2**	0.0347***	0.0313***	0.0337***	0.0198*
	(3.0822)	(2.7068)	(2.8631)	(1.7754)
**TS**		-0.0323	-0.0235	0.1012
		(-1.3536)	(-1.0091)	(1.1253)
**Fin**		-0.0295*	-0.0350**	0.0348
		(-1.7099)	(-1.9950)	(1.2489)
**lnFDI**		0.0596***	0.0445***	-0.0169
		(3.3502)	(2.9116)	(-1.3240)
**Rmd**		-0.0179	0.0399	-0.0272
		(-0.2207)	(0.4233)	(-0.1804)
**Ur**		1.2010***	1.3043***	1.7580***
		(6.6608)	(6.4850)	(3.7884)
**Hz**		0.1617**	0.1895***	-0.3670***
		(2.5718)	(3.0794)	(-3.6670)
**Province**	No	No	No	Yes
**Year**	No	No	Yes	Yes
**_Cons**	4.6783***	3.4240***	3.1828***	7.2344***
	(10.8641)	(6.0796)	(6.2059)	(7.5527)
**N**	330	330	330	330
**R** ^**2**^	0.0875	0.4259	0.4354	0.9339

Note: t-values in parentheses

* p < 0.1

** p < 0.05

*** p < 0.01.

From the table, we observe that, regardless of whether control variables are included, the coefficient of the entrepreneurship level is significantly negative, at least at the 5% level, and the coefficient of the quadratic term is significantly positive, at least at the 10% level. This indicates that the impact of entrepreneurship on common prosperity follows a U-shaped relationship. Specifically, when the entrepreneurial group is small, entrepreneurship activities may lead to a few individuals becoming wealthy first, increasing income disparity and hindering common prosperity. However, entrepreneurship promotes common prosperity as the entrepreneurial group reaches a certain size.

### Heterogeneity analysis

#### 4.4.1. Heterogeneity analysis of different regions

In China, significant differences exist in resource endowments, development types, and levels between the northern and southern regions, leading to evident regional heterogeneity in innovation, entrepreneurship, and common prosperity. Therefore, the impact of innovation and entrepreneurship on common prosperity may also exhibit regional heterogeneity. We divided the 30 provinces (autonomous regions and municipalities) into northern and southern regions for regression analysis, and the results are presented in Table 7.

**Table 7:** Heterogeneity test (by different region).

Variables	Northern regions	Southern regions	Northern regions	Southern regions
	Cpi	Cpi	Cpi	Cpi
**Ino**	0.1175***	0.0389		
	(3.1419)	(0.9256)		
**Entre**			-0.0165	-0.2131**
			(-0.5102)	(-2.1974)
**TS**	0.1890***	1.5270	0.0850	0.8179
	(2.7944)	(1.4621)	(1.4291)	(0.9461)
**Fin**	0.0403	-0.0041	0.0417	-0.0083
	(1.5038)	(-0.0643)	(1.4621)	(-0.1380)
**FDI**	-0.0147*	-0.0921**	-0.0084	-0.0791**
	(-1.9233)	(-2.4438)	(-1.0587)	(-2.4128)
**Rmd**	-0.1693*	0.5241	-0.2001**	0.6554
	(-1.7923)	(0.9875)	(-2.0782)	(1.2608)
**Ur**	1.3759***	0.8569	1.4277***	0.7508
	(3.3261)	(1.2878)	(3.3548)	(1.0539)
**Hz**	-0.1809	-0.3046	-0.0788	-0.2868
	(-1.5548)	(-1.3649)	(-0.6653)	(-1.3852)
**Province**	Yes	Yes	Yes	Yes
**Year**	Yes	Yes	Yes	Yes
**_Cons**	3.0639***	2.1832	3.5900***	5.5415**
	(3.9584)	(0.8899)	(4.5583)	(2.1485)
**N**	165	165	165	165
**R** ^**2**^	0.9380	0.9411	0.9341	0.9440

Note: t-values in parentheses

* p < 0.1

** p < 0.05

*** p < 0.01.

According to models (1) and (2) in Table 7, the coefficient of innovation on common prosperity is significantly positive in the northern region but insignificant in the southern region. This indicates that innovation promotes common prosperity in the northern region, whereas its effect is insignificant in the southern region. In contrast, according to models (3) and (4) in Table 7, the coefficient of entrepreneurship on common prosperity is significantly negative in the southern region but not significant in the northern region. This implies that entrepreneurship significantly impacts common prosperity in the southern region, whereas its effect on common prosperity in the northern region is insignificant. These regional differences may be attributed to variations in the industrial composition between the northern and southern regions.

Due to resources and geographical location, China’s industrial layout exhibits regional characteristics, focusing on heavy industries in the north and relatively developed light industries in the south. Heavy industries heavily rely on technological innovation for development, leading to relatively higher investment in this aspect by enterprises. On the other hand, entrepreneurship in heavy industries requires substantial capital and poses greater challenges, while entrepreneurship in light industries is comparatively easier. Moreover, the southern regions are more flexible in thinking and exhibit higher entrepreneurial activity, significantly influencing common prosperity. Conversely, the northern regions are more impacted by innovation.

#### 4.4.2. Heterogeneity analysis of different economic development level

In general, differences exist in the level of economic development among different regions, leading to variations in the baseline effects of innovation and entrepreneurship, which in turn influence common prosperity differently. In this study, we measure the regional economic development level using Gross Domestic Product (GDP) and divide the sample into regions with high and low economic development levels for conducting grouped regression analysis. The empirical results are presented in Table 8.

**Table 8:** Heterogeneity test (by different economic development levels).

Variables	High Economic Development Level	Low Economic Development Level	High Economic Development Level	Low Economic Development Level
	Cpi	Cpi	Cpi	Cpi
**Ino**	-0.0142	0.1681**		
	(-0.3989)	(2.5308)		
**Entre**			-0.0096	-0.1859*
			(-0.2565)	(-1.9702)
**TS**	-0.2784	0.2660**	-0.2620	0.1873*
	(-0.8982)	(2.4863)	(-0.8254)	(1.8958)
**Fin**	0.0219	0.0145	0.0230	0.0190
	(0.6177)	(0.3697)	(0.6746)	(0.4777)
**FDI**	-0.0029	-0.0319**	-0.0030	-0.0243*
	(-0.2165)	(-2.2855)	(-0.2144)	(-1.7187)
**Rmd**	-0.2408	-0.0630	-0.2263	-0.0370
	(-1.1329)	(-0.3831)	(-0.9955)	(-0.2121)
**Ur**	1.0394**	1.2380	0.9454**	2.4780**
	(2.5234)	(1.5569)	(2.4516)	(2.5198)
**Hz**	0.0471	-0.6916**	0.0587	-0.5132*
	(0.4249)	(-2.0483)	(0.5335)	(-1.8142)
**Province**	Yes	Yes	Yes	Yes
**Year**	Yes	Yes	Yes	Yes
**_Cons**	3.9573***	7.2363***	3.8105***	7.0436***
	(3.6316)	(3.1828)	(3.7392)	(3.1422)
**N**	154	176	154	176
**R** ^**2**^	0.9171	0.9295	0.9170	0.9284

Note: t-values in parentheses

* p < 0.1

** p < 0.05

*** p < 0.01.

According to model (1)(2) in Table 8, the impact coefficient of technological innovation on common prosperity is significantly positive in regions with a low economic development level but not significant in regions with a high economic development level. This indicates that innovation can effectively drive common prosperity in regions with lower economic development levels but does not significantly impact common prosperity in regions with higher economic development levels. This disparity may be attributed to the low technological level and limited technology diffusion in regions with lower economic development levels. This results in innovation’s more pronounced positive effect on total factor productivity in less developed areas. This can help reduce the technological gap between urban and rural areas and between developed and underdeveloped regions, thus increasing residents’ income while narrowing the income disparity among different groups and regions.

Similarly, according to Model (3)(4) in Table 8, the impact coefficient of entrepreneurship on common prosperity is significantly negative in regions with a low economic development level but not significant in regions with a high economic development level. This suggests that entrepreneurial activities negatively impact common prosperity in regions with lower economic development levels while not significantly affecting common prosperity in regions with higher economic development levels. This difference may arise from the more active entrepreneurial environment and diverse policy support for entrepreneurship in regions with higher economic development levels, leading to a larger population engaged in entrepreneurial activities and being closer to the goal of common prosperity. In contrast, regions with lower economic development levels have a smaller population of entrepreneurs, and even successful entrepreneurs may still represent a small proportion of the population, resulting in a larger wealth gap. Additionally, regions with higher economic development levels generally have more high-paying job opportunities, higher average wages for ordinary workers, and a smaller income gap between wage earners and the entrepreneurial class than regions with lower economic development levels.

#### 4.4.3. Heterogeneity analysis of different fixed capital stock level

The accumulation of fixed capital in the entire society provides the material foundation for achieving common prosperity. The fixed capital stock level also influences the role of innovation and entrepreneurship in achieving common prosperity. We calculate the initial capital stock for each province in 2001, using the total fixed capital formation in that year divided by the average depreciation rate of 10.96% plus the average investment growth rate from 2001 to 2005 [64]. We then use this initial capital stock to calculate the current capital stock based on the formula: current capital stock = previous capital stock×(1–10.96%) + current total fixed asset formation. After grouping the data according to the calculated fixed capital stock levels, we conducted a regression analysis, and the results are presented in Table 9.

**Table 9:** Heterogeneity test (by different fixed capital stock).

Variables	High Fixed Capital Stock	Low Fixed Capital Stock	High Fixed Capital Stock	Low Fixed Capital Stock
	Cpi	Cpi	Cpi	Cpi
**Ino**	-0.0068	0.1102**		
	(-0.1176)	(2.2477)		
**Entre**			0.0115	-0.1888**
			(0.2859)	(-2.0538)
**TS**	0.0572	0.1936**	0.0771	0.1668*
	(0.1520)	(2.0010)	(0.1988)	(1.7648)
**Fin**	0.0265	0.0369	0.0289	0.0301
	(0.8172)	(0.9870)	(0.9318)	(0.8082)
**FDI**	-0.0040	-0.0360**	-0.0058	-0.0189
	(-0.2178)	(-2.5888)	(-0.3140)	(-1.4795)
**Rmd**	-0.0505	-0.0110	-0.0664	-0.0690
	(-0.1749)	(-0.0597)	(-0.2245)	(-0.4068)
**Ur**	1.3428**	1.5479***	1.3613**	1.7788***
	(2.3649)	(2.7196)	(2.4768)	(2.8670)
**Hz**	-0.0181	-0.5459*	-0.0184	-0.4017
	(-0.1486)	(-1.7437)	(-0.1532)	(-1.5663)
**Province**	Yes	Yes	Yes	Yes
**Year**	Yes	Yes	Yes	Yes
**_Cons**	3.3317**	6.2542***	3.1698***	7.0589***
	(2.4761)	(3.1860)	(2.6481)	(3.2149)
**N**	121	209	121	209
**R** ^**2**^	0.8425	0.9356	0.8427	0.9365

Note: t-values in parentheses

* p < 0.1

** p < 0.05

*** p < 0.01.

According to Models (1) and (2) in Table 9, the coefficient of innovation is significant and positive in regions with low fixed capital stock. At the same time, it is not significant in regions with high fixed capital stock. This suggests that innovation significantly promotes common prosperity in regions with lower fixed capital stock but has yet to significantly impact common prosperity in regions with higher fixed capital stock. This difference might be attributed to the fact that in regions with higher fixed capital stock, the "cake" is relatively larger, and the positive effect of innovation on expanding the "cake" is less pronounced compared to regions with lower fixed capital stock. Additionally, in regions with higher fixed capital stock, the industries are relatively fixed, making narrowing the income gap between different population groups challenging.

Similarly, based on Models (3) and (4) in Table 9, the coefficient of entrepreneurship is not significant in regions with high fixed capital stock. At the same time, it is significant and negative in regions with low fixed capital stock. This observation suggests that entrepreneurship is yet to exhibit a substantial influence on shared prosperity within regions characterized by a higher level of fixed capital stock; however, its impact appears adverse when considering regions marked by a lower level of fixed capital stock and their pursuit of common prosperity. The reason for this difference might be that in regions with higher fixed capital stock, the promoting effect of entrepreneurship on prosperity outweighs its adverse effect on equality, whereas in regions with lower fixed capital stock, entrepreneurship activities widen the income gap between different population groups, showing a negative impact on common prosperity.

### Moderating effects analysis

Government fiscal and market mechanisms play a crucial role in promoting the realization of common prosperity and also moderate the impact of innovation and entrepreneurship on common prosperity. Based on econometric models (2)-(5), we examine the moderating effects of government fiscal policies and market mechanisms on the relationship between innovation, entrepreneurship, and common prosperity. Table 10 presents the regression results. Model (1) investigates the moderating effect of government fiscal policies on the relationship between innovation and common prosperity. Model (2) examines the moderating effect of market mechanisms on the relationship between innovation and common prosperity. Model (3) explores the moderating effect of government fiscal policies on the relationship between entrepreneurship and common prosperity. Model (4) analyzes the moderating effect of market mechanisms on the relationship between entrepreneurship and common prosperity.

**Table 10:** Moderating effects of government fiscal policies and market mechanisms.

Variables	Cpi	Cpi	Cpi	Cpi
	(1)	(2)	(3)	(4)
**Ino**	0.0573*	0.0857**		
	(1.6902)	(2.4770)		
**Entre**			-0.1079***	-0.1107***
			(-2.7323)	(-2.7135)
**Gov**	7.5211*		1.8244	
	(1.9260)		(0.5710)	
**Mar**		-0.0090		-0.0007
		(-0.5315)		(-0.0487)
**Ino_Gov**	-3.2010***			
	(-2.9196)			
**Ino_Mar**		-0.0034		
		(-0.8043)		
**Entre_Gov**			-4.3909**	
			(-2.1297)	
**Entre_Mar**				0.0095*
				(1.6983)
**Controls**	Yes	Yes	Yes	Yes
**Province**	Yes	Yes	Yes	Yes
**Year**	Yes	Yes	Yes	Yes
**_Cons**	Yes	Yes	Yes	Yes
**N**	330	330	330	330
**R** ^**2**^	0.9347	0.9331	0.9343	0.9339

Note: t-values in parentheses

* p < 0.1

** p < 0.05

*** p < 0.01.

In Model (1), the coefficient of the interaction term between innovation and government fiscal variables is significantly negative at the 1% level. Model (1) indicates that government fiscal policies weaken the positive effect of innovation on common prosperity, showing a significant negative moderating effect. When the degree of government fiscal policy regulation is low, the positive impact of innovation on achieving common prosperity is more pronounced. However, as the degree of government fiscal policy regulation increases, the positive effect of innovation gradually diminishes, suggesting a clear substitutive relationship between innovation and government fiscal policy regulation in promoting common prosperity. In Model (2), the coefficient of the interaction term between innovation and the degree of marketization is not statistically significant. This indicates that the degree of marketization does not significantly moderate the relationship between innovation and common prosperity.

In Model (3), the coefficient of the interaction term between entrepreneurship and government fiscal variables is significant at the 5% level. This suggests that government fiscal policies reinforce the adverse impact of entrepreneurship on common prosperity. The income tax collected by the government may be used for entrepreneurial support subsidies to provide initial funding for entrepreneurs and enhance entrepreneurial enthusiasm, aiming to increase the middle-income group and reduce the wealth gap between different groups. However, due to information asymmetry and other factors, the government’s entrepreneurial funding may not be effectively directed to those in genuine need. This leads to a phenomenon where the rich become richer, and the poor become poorer.

In Model (4), the coefficient of the interaction term between entrepreneurship and marketization degree is significant at the 10% level. This indicates that market mechanisms weaken the negative impact of entrepreneurship on common prosperity. Under market mechanisms, the proactive behavior of enterprises and individuals can be mobilized, leading to increased efficiency and prosperity. As prosperity increases, the overall supply of distributable products also grows. Additionally, in a market-driven environment, individuals engaged in independent entrepreneurship can adapt their production based on market demand and consumer preferences, enabling more efficient resource utilization and improving the supply of distributable products.

## Conclusions

Common prosperity is an essential requirement of socialism with Chinese characteristics. The central government has elevated it to a strategic level and placed it in a prominent position, searching for the path to achieving common prosperity of significant importance. Based on balanced panel data from 30 provinces and direct-controlled municipalities (excluding Hong Kong, Macau, Taiwan, and Tibet) for 2010–2020, this study empirically examines the impact of innovation and entrepreneurship on common prosperity. The findings indicate that innovation significantly promotes common prosperity [65]. However, the scale of entrepreneurship may have a negative impact on common prosperity when it has yet to reach a certain level. Conversely, when the scale of entrepreneurship reaches a certain threshold, it can positively affect common prosperity. This result differs from the conclusions of most existing literature [44], which suggests that entrepreneurship significantly promotes common prosperity.

Heterogeneity tests have revealed that the impact of innovation on common prosperity is significant in northern regions, while the impact of entrepreneurship on common prosperity is significant in southern regions, indicating regional heterogeneity. Additionally, innovation and entrepreneurship significantly impact common prosperity in regions with low economic development levels and low fixed capital stock.

Through the analysis of moderating effects, it is evident that leveraging market mechanisms and effective government functions is crucial in promoting common prosperity. Market and government play important moderating roles in the impact of innovation and entrepreneurship on common prosperity. Government fiscal policies weaken the positive effect of innovation on common prosperity, indicating a substitutive relationship between the two. On the other hand, concerning the impact of entrepreneurship on common prosperity, government fiscal policies strengthen its influence, while market mechanisms attenuate its effect.
